# Increased impact sensitivity in ageing high explosives; analysis of Amatol extracted from explosive remnants of war

**DOI:** 10.1098/rsos.231344

**Published:** 2024-03-27

**Authors:** Geir P. Novik, Dennis Christensen

**Affiliations:** ^1^ Department of Safety, Economics and Planning, Faculty of Science and Technology, University of Stavanger, P.O. Box 8600, Stavanger 4036, Norway; ^2^ Norwegian Defence Research Establishment (FFI), P.O. Box 25, Kjeller 2027, Norway; ^3^ Department of Mathematics, University of Oslo, P.O. Box 1053 Blindern, Oslo 0316, Norway

**Keywords:** unexploded ordnance, ERW, the bruceton method, sensitivity analysis, high explosives

## Abstract

Millions of tonnes of explosive remnants of war remain in nature and their volume is continuously growing. The explosive legacy of wars represents an increasing threat to the environment and societal safety and security. As munitions continue to deteriorate, harmful constituents will eventually leak into the environment, poisoning ecological receptors and contaminating the surrounding soil and groundwater. Moreover, munition deterioration due to exposure to various environmental factors may ultimately cause them to become increasingly sensitive to external stimuli and susceptible to accidental detonation. To thoroughly assess how to address these ageing munitions, we must first establish certain threshold values for safe and secure handling and final disposal of the explosive ordnance. One key factor is to establish how the impact sensitivity of the explosives evolves over time. In the present work, we investigated the high-explosive substance Amatol extracted from ageing explosive remnants of war. The results obtained in the analysis indicate that the high explosives in the examined specimens were generally much more sensitive to impact than previously assumed. Furthermore, the analysis revealed that the standardized methodology of impact sensitivity testing was insufficient for estimating the sensitivities in question, and a more careful statistical analysis is required.

## 1. Introduction

During the Shell Crisis of 1915, the stock of UK artillery shells was unexpectedly depleted due to an unanticipated and prolonged period with a high rate of fire on the front lines in World War I (WWI). It soon became evident that the supply of high explosives in use (predominantly 2,4,6-trinitrotoluene (TNT) and 2,4,6-trinitrophenol (picric acid)) was insufficient [1]. To eke out the available supply of TNT for shell, grenade and bomb fillings, the Research Department at the Royal Arsenal in Woolwich developed mixtures of ammonium nitrate and TNT. These binary mixtures, known as Amatols, were easy to manufacture and exhibited several favourable properties, including the effectiveness they exhibited in shell-bursting trials. Ammonium nitrate, which was manufactured from atmospheric nitrogen for the first time, was a readily available explosive ingredient and more valuable since it leaves no solid residues upon decomposition and ensures a high volume of gaseous explosion products [2]. Live fire gun trials substantiated the trials at rest, and the adoption of Amatols as high-explosive fillings in munitions followed quickly thereafter [3].

In addition to being an easily available explosive in times of necessity, Amatols also enabled a highly economical output of explosive materials, as the cost of ammonium nitrate was about one-quarter of that of TNT. Amatols were therefore proposed to economize the volume of TNT and simultaneously take advantage of the excess oxygen present in ammonium nitrate to compensate partially or completely for TNT’s oxygen deficiency [4]. For similar reasons, several governments authorized its use shortly after Great Britain (e.g. [5]).

TNT and Amatols were the preferred high-explosive fillings for most high-explosive artillery shells at the outset of World War II (WWII), largely due to their availability and combination of high power and low sensitivity. In particular, they were easy and safe to handle and transport. Towards the end of WWII, the rapid production of an enormous amount of TNT eventually removed the necessity of using ammonium nitrate as a substitute for TNT. Another contributing factor to the disuse of Amatols as high explosives in munitions was the emergence of other explosives during WWII, such as pentaerythritol tetranitrate (PETN) and cyclotrimethylenetrinitramine (RDX) and their binary and ternary mixtures, which are more powerful than TNT [6].

Although they are now mostly obsolescent, Amatols were universally used for several decades by all nations in all types of ammunition as a substitute for TNT [6]. Consequently, the only time Amatols are normally encountered in explosive ordnance today is in legacy munitions, at ammunition dumping sites and in explosive remnants of war (ERW).

Since a considerable percentage of both WWI and WWII munitions contained Amatol filling, understanding its ageing characteristics is a subject of immense importance. Several studies have revealed that the deterioration of explosive fillers can make the munitions increasingly sensitive to external stimuli and susceptible to detonation when exposed to heat, shock or friction [7–10]. Moreover, an increasing number of spontaneous detonations have been reported in ageing munitions, possibly due to deteriorating or changing technical or chemical properties [11,12]. Previous studies regarding samples of high explosives extracted from ERW (e.g. TNT and PETN) have indicated that the impact sensitivity of ageing explosives does not appear to have been reduced over the last eight decades, and in some cases, the explosives can even become more sensitive to stress [13]. Some reports have also indicated that under specific circumstances, Amatols can form dangerous compounds that may increase their sensitivity (i.e. [5,6,14]). However, few studies have analysed the properties of ageing Amatols in ERW. Consequently, we do not have sufficient data available to properly assess the risks related to spontaneous detonation or the clearance and handling of ERW with the Amatol filling.

ERW at terrestrial and aquatic sites also present an international environmental problem due to the release of explosive materials from corroding ordnance, in addition to the risks associated with the potential for accidental detonations [15]. Similar to most explosive fillings used in munitions, Amatols represent a source of contamination that can be toxic to ecological receptors, causing damage to impacted sites and surrounding areas exposed to the offsite migration of contaminants. As many of the chemicals used in ammunition are highly poisonous and have proven to affect living organisms and contaminate the surrounding soil and groundwater, the leakage and bioaccumulation of toxic constituents from corrosive munitions pose a formidable threat to the ecosystem [16–20]. Some constituents of munitions have also been proven to enter the food chain and could, therefore, directly affect human health through the consumption of contaminated food [21].

As munition casings continue to deteriorate, we expect an increase in the release of their harmful constituents in the future [22]. Consequently, there is a time constraint regarding the safe and appropriate identification and handling of ERW and their explosive fillings based on an assessment of the viable options. As a result of the potential hazards related to ERW risks, their removal is a highly prioritized task for many countries and international organizations, such as the North Atlantic Treaty Organization (NATO) and the United Nations (UN) [23]. To properly assess and ideally mitigate the risks related to accidental detonations and the uncontrolled release of harmful substances, we rely on accurate data and a proper statistical analysis of the specifics of the applicable ERW, including its sensitivity.

In this study, we have analysed the impact sensitivity of Amatol extracted from ageing ERW via statistical analyses of new fallhammer measurements. Following the recommendations of Christensen *et al*. [24], we employed the Bruceton up-and-down test procedure and computed confidence intervals using Fieller’s theorem. Our analysis reveals that all collected samples exhibited higher sensitivity than the standard reported value for Amatol in the literature. This study, therefore, also serves to illustrate why these standards are insufficient and require an update.

## 2. Material and methods

### 2.1. Sample characteristics

The first experiments using ammonium nitrate (H_4_N_2_O_3_) as a component in explosive mixtures began in the second half of the nineteenth century, although the substance was originally discovered 200 years earlier [25]. However, it only gained supreme military importance as an ingredient of high explosives during WWI [4]. One of the most commonly used military high explosives at the outbreak of WWI was TNT (C_7_H_5_N_3_O_6_). This was partially due to its explosive characteristics (i.e. high output and low sensitivity) and also because of its ease of manufacture and suitability for melt loading, either as a pure explosive or as a binary mixture [26]. Since the colossal demand for high explosives in WWI could not be fulfilled by the output of explosives such as TNT and picric acid, various compositions, such as mixtures of aromatic compounds with ammonium nitrate, were introduced and widely implemented [2]. These compositions involved mixing of two or more explosive compounds to produce explosive substances with more suitable characteristics. Generally, the properties of these compositions exhibit an intermediate state between those of the individual explosive ingredients [27].

**Figure 1.**
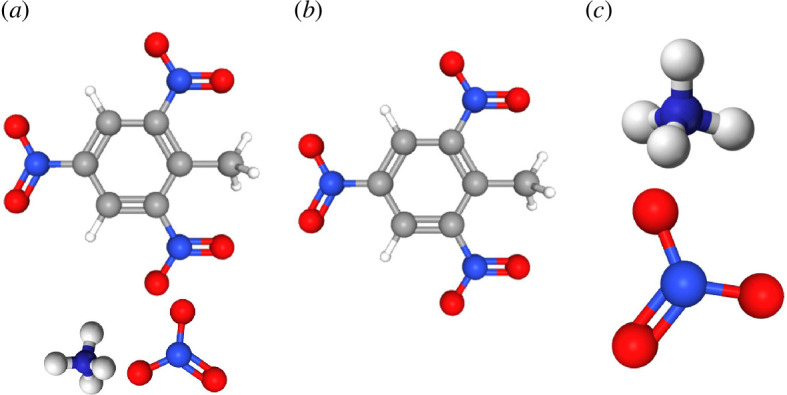
Chemical formulas of (*a*) Amatol and its constituents; (*b*) TNT; and (*c*) ammonium nitrate [28].

Amatols (C_7_H_9_N_5_O_9_) are binary mixtures of ammonium nitrate and TNT, as illustrated in figure 1. Compared to TNT, they were cheaper to produce and produced greater volumes of gas per unit weight upon explosion [1]. When TNT detonates, free carbon is present, indicating that it is deficient in oxygen [4]. On the other hand, the addition of ammonium nitrate to Amatols, which is rich in oxygen, results in a more complete combustion of the TNT component. As a result, the smoke produced by the detonation of Amatol has a light white-yellowish colour, in contrast to the heavy black smoke produced by the detonation of pure TNT [1]. In general, due to its tendency to increase chemical stability and decrease sensitivity to friction and shock, ammonium nitrate is the most widely used oxygen carrier in explosives [25]. Although it is technically possible to detonate straight ammonium nitrate with a sufficiently powerful impulse, its chemical properties suggest that it should not be used alone as an explosive [4].

There are many types of Amatols, which differ only in terms of the proportion at which TNT and ammonium nitrate are present [4]. The composition of any one of these is reflected in its nomenclature. Thus, *Amatol 80/20* denotes a mixture of 80% ammonium nitrate with 20% TNT by mass. Generally, the first number invariably refers to the percentage of ammonium nitrate, although in the German nomenclature, Amatol compositions (known as various types of Füllpulver, abbreviated as Fp. 60/40, 20/80, etc.), the numerators refer to the percentage of TNT present. The principal Amatols are 40/60 and 80/20. Examples of other proportions that have been used are 45/55, 50/50, 83/17 and 90/10.

Amatols were widely used during WWI and WWII in many countries. In particular, the mixture consisting of 40% ammonium nitrate and 60% TNT accrued immense importance [2]. In Germany, it was known as Füllpulver No 13 or Fp. 60/40 and in Great Britain, Amatol 40/60, and it was cast-loaded into a wide variety of bombs and shells [29]. However, as Amatols are generally considered comparatively insensitive, they require a special exploder system to ensure complete detonation [4]. When efficiently detonated, Amatol 40/60 is slightly less powerful than TNT alone. Owing to the hygroscopic nature of ammonium nitrate, Amatols are considered highly unstable in storage, unless it is possible to exclude moisture. For example, at 90% relative humidity (RH) and 30°C, Amatol 80/20 could contain approximately 61% moisture within two days. This would not only reduce the sensitivity and velocity of the detonation to a low order but could also result in a failure to detonate [1]. Another effect that has been observed as a result of exposure to moisture and high temperatures is that Amatol may congeal into a dense, hard mass as a result of changes in the crystalline form of ammonium nitrate [4].

In the existing literature, Amatols are generally considered to be equally or less sensitive to impact than TNT (e.g. [1,4–6,27,30,31]). However, some reports suggest that the introduction of impurities into the production of Amatol can result in slightly increased sensitivity compared to pure TNT [4]. According to a study by Hackel (1937, as cited in [2]), the impact sensitivity of mixtures of nitro compounds with ammonium nitrate (Amatols) was found to be higher than that for pure nitro compounds due to the friction produced by the hard crystals of ammonium nitrate. In this study, Hackel found mixtures containing 30 to 60% of ammonium nitrate to be equally as sensitive as picric acid, an explosive substance that is slightly more sensitive to impact than TNT [6]. However, due to the hygroscopic nature of ammonium nitrate, it will begin to deteriorate when exposed to water, and studies have demonstrated that the explosive compositions containing ammonium nitrate can become progressively less sensitive to impact as the moisture content increases [32]. It has also been proven that the impact sensitivity can be reduced to a level where the amount of force required for the initiation of the substances makes them impracticable as explosives (e.g. [33]), as standard means of initiation would result in failures to detonate [1]. Moreover, a high moisture content can decrease detonation velocity, which, in many cases, makes the continuation of the explosive shockwave within the substance unachievable by its own means.

However, studies have also revealed that the presence of moisture, along with other factors, can contribute to an increase in the impact sensitivity of Amatols. It is known that explosive compositions containing ammonium nitrate may become sensitized when contaminated with small amounts of metals or when they come into contact with metals. These contaminating metals may react chemically with ammonium nitrate, forming complex salts and sensitizing the mixture [32]. The contamination of Amatols could occur during normal handling and mixing, or they could come into contact with bare metal surfaces when loaded into ordnance or if any preventive lacquers deteriorate over time. An investigation of the stability of various mixtures of ammonium nitrate and TNT conducted after WWII at the Laboratoire Centrale des Poudres in Paris also demonstrated that mixtures of military-grade TNT and pure ammonium nitrate had, in some cases, decomposed with the evolution of ammonia that attacked TNT to form various unstable coloured compounds (F. M. Lang and J. Boileau, 1952, as cited in [1]). According to Fedoroff *et al*. [1], in the presence of iron, the hydrolysis of moist ammonium nitrate may occur with the formation of ammonia solution (NH_4_OH), which reacts with TNT to form an exudate of a brown oily material igniting at 67°C. This can be detected by the discolouration of the explosive and the odour of ammonia (NH_3_). In addition to being reactive to iron, mixtures of Amatols may, in the presence of moisture, also react with metals such as copper, brass, bronze and lead, forming dangerously sensitive compounds with copper and its alloys [6,14]. However, since this was a well-known attribute of Amatols, it was considered general practice at the time to coat the insides of munitions with acid-proof paint prior to loading to prevent corrosion caused by contact between Amatols and metals [1].

### 2.2. Sampling location and methodology

**Figure 2.**
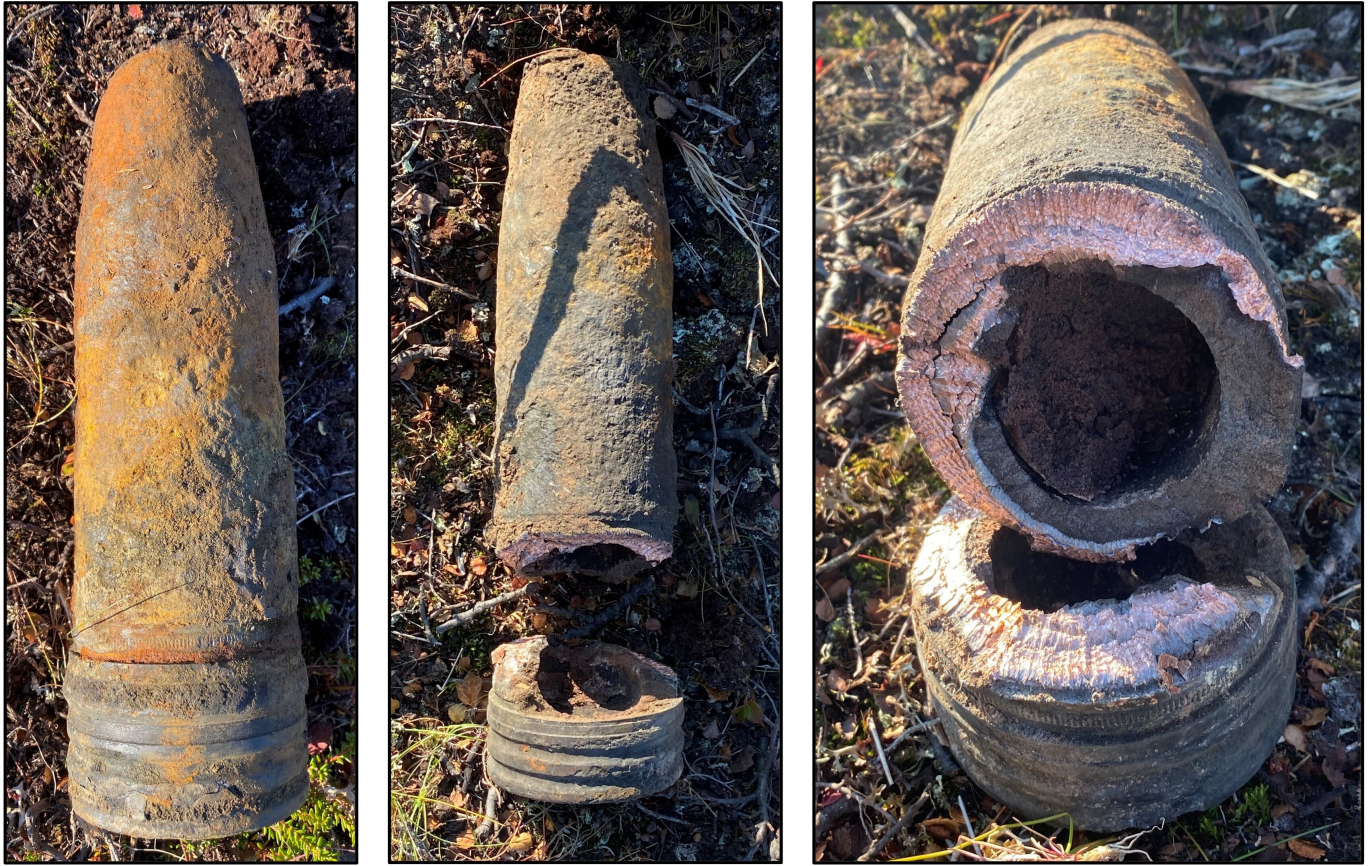
A German 88 mm HE projectile (type 8.8 cm Sprgr. Patr. L/4.5 (Kz)) cut with a flexible linear-shaped charge. Its filling was identified as Füllpulver 60/40 (i.e. Amatol 40/60).

To ensure the reliability of the data, all samples of Amatol in this study were extracted from live ordnance originating from WWII during national explosive ordnance disposal (EOD) clearance operations in Norway. Consequently, all explosive objects used in this analysis originate from explosive ordnance that was produced before May 1945. All the explosive objects were localized and reported to the relevant governmental agencies by members of the public before their exploitation and final disposal. In all cases, the munitions were subjected to handling (moving the object) by the discoverer or by the designated EOD team. All the samples of high explosives were extracted from relevant objects and analysed within the last three years (2021–2023). The first author personally conducted the physical extraction of the high explosives from the ordnance. In all situations, it was determined to be safe to move the explosive objects to a site suitable for the extraction of high-explosive samples as well as for the final disposal of the ordnance. All explosive objects included in this study are of German origin and were located in an area heavily contaminated with explosive remnants of WWII, namely Finnmark County in the northernmost region of Norway. The required disassembly of the ordnance to gain access to their high explosive fillings was performed with the use of explosive cutting charges (shaped charges), as indicated in the example in figure 2.

After the required dismantling of the explosive ordnance, an initial sample of high explosives was retrieved from the point of entry, specifically the centre mass of the explosives. Since some of the studied objects were found to contain several types of high-explosive fillings, multiple samples were collected from various compounds (in the case of Amatol fillings, casted TNT was frequently used as a seal to prevent any moisture from coming into contact with the hygroscopic Amatols).

In total, high-explosive samples were collected from over 20 unexploded objects potentially containing Amatol fillings. Among these, five unique samples of Amatol were included in this study. Of these five, three were discovered with their respective fuzes installed and (based on a visual inspection) they appeared to be still fully intact (i.e. no visible cracks or fractures in their outer casings). The remaining two objects were also apparently intact but were found without fuzes installed, increasing the exposure of their explosive fillings to environmental factors. Of these two, one was retrieved from an ammunition dumping site (lake) at about a five-metre depth. The remaining four objects were all located on land. The specifics for the particular objects are as follows: one German HE artillery projectile (no fuze, located in water (hereafter designated as substance A)), one German HE aerial bomb (no fuze, located on land (substance B)), one German HE artillery projectile (fuze installed, located on land (substance C)), two German HE mortar projectiles (fuze installed, located on land (substances D and E, respectively)).

According to the relevant literature, all ordnances included in this study were identified as carrying high-explosive fillings of the substance Füllpulver 60/40, otherwise known as Amatol 40/60 [34–38]. The identification of Amatols was later confirmed by analysing the samples with an ion chromatograph and an ultra-performance liquid chromatography-mass spectrometer (UPLC-MS/MS).

### 2.3. Storage and preparation of samples

After extraction, the Amatol samples were immediately placed in airtight containers (50 ml sterile polypropylene screw-cap tube) and stored in approved ammunition storage facilities. Apart from humidity control (at a maximum of 50% RH), the samples were stored under normal atmospheric conditions, with temperature fluctuations similar to those appearing in nature, albeit with less violent variations, as the samples were stored under cover and protected from direct sunlight.

The physical appearance of all samples was found to resemble a grainy, brown sugar-like form (as opposed to the white to light buff colour normally associated with Amatol), indicating the presence of impurities in the composition or that the Amatols had been exposed to light and moisture [6].

At the time of extraction, all substances, except for substance A, appeared to be dry and powdery. We determined the exact moisture content of each sample as follows: first, we introduced a dried Pyrex crystallizer with a ribbed cover, with combined mass W_1_ (all masses were accurate up to 1/10 mg). The ribbed cover was used to catch the small amounts of TNT that sublime upon heating [1]. Each substance was then analysed by adding a sample of mass $W_{S}$ to the crystallizer. The total mass of the crystallizer, cover and sample was obtained, and the specimen was heated for 2 to 3 hours at a temperature of 75°C and then cooled in a desiccator. With $W_{2}$ as the combined mass of the specimen after this process, the original moisture content w (as a percentage) of the sample is yielded by


$$w=100\left\{W_{S}-\left(W_{2}-W_{1}\right)\right\}/W_{S}.$$

It was found that the moisture content of substance A was 22.2% and the remaining substances had moisture contents of 0.33 ± 0.25%. These results coincide with the individual physical appearances of the samples at the time of extraction. However, their discolouration indicates that all of the tested substances may have undergone some exposure to moisture at one point in time.

Prior to the impact sensitivity analysis, the samples were prepared in accordance with the requirements of NATO STANAG 4489 [39] and the United Nations Manual of Tests and Criteria - Classification Procedures, Test Methods and Criteria Relating to Explosives, Test 3 (a) (ii) [40]. Powdered substances are to be sieved, and only the fraction with a particle size of 0.5–1.0 mm is to be used for testing. For pressed or cast substances, where their powder is excessively coarse to pass the sieve, their particle sizes are reduced by gently crushing them using a pestle and mortar. Only the fraction that passed a 1000 µm sieve and retained on a 500 µm sieve was used for the test.

As one of the substances consisted of a paste-like material (substance A), it was treated as a *paste-like or gel-type substance* as per ([40], p. 86) test procedures, wherein a cylindrical tube of 40 mm^3^ capacity (3.7 mm diameter and 3.7 mm height) is inserted into the substance and, after levelling off the surplus, the sample is removed from the tube using a wooden rod. A sample from this substance was placed in a humidity-controlled environment to reduce the moisture level of the sample to about 0.5% in preparation for further analysis. This particular substance was analysed in both its original (22.2% moisture) and prepared (0.33% moisture) states, hereafter denoted as substance A_1_ (original) and substance A_2_ (prepared), respectively.

### 2.4. Impact sensitivity testing

The impact sensitivity of an explosive substance refers to its susceptibility to detonation upon impact. This parameter characterizes the safety of explosives in handling and transportation [41]. To determine the impact sensitivity of a substance, a device known as fallhammer apparatus is normally used. There are several versions of these types of devices, but the United Nations recommends the Bundesanstalt für Materialforschung und -prüfung (BAM) fallhammer, which has become the most frequently used standard impact sensitivity measuring device [42]. However, the various apparatuses all operate on the same principle: a sample of assorted sizes of the tested explosive substance are subjected to the impact of falling weights, and the researcher estimates the sensitivity of the explosive based on which heights resulted in explosions [43]. The main differences between the various fallhammer apparatuses are mainly related to their design and the manner in which the sample is subjected to the drop weight impact via different types of plungers [44]. It is currently an active area of research to better understand how energy is transferred through the explosive sample in the fallhammer test (e.g., [45,46]).

**Figure 3.**
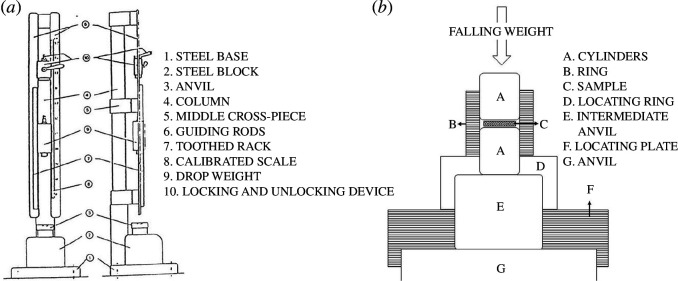
(*a*) The BAM impact machine [39] and (*b*) the fallhammer impact device [39].

The BAM fallhammer test was initially developed to obtain better reproducible data compared to existing tests at that time [43] and is generally considered to yield reasonably reproducible results [44]. In this analysis, the OZM BFH 12 BAM Impact Apparatus was applied, and the tests were performed in accordance with the requirements of the test procedure described in NATO STANAG 4489, Annex C; BAM Impact Machine [39]. The BAM Impact Machine is presented in figure 3*a*. The essential parts of the BAM fallhammer are the steel block with the base, the anvil, the guiding rods, the drop weight with the locking and unlocking device and the impact device. The impact device, as presented in figure 3*b*, consists of two coaxially arranged steel cylinders with polished surfaces and rounded edges, held in place by a cylindrical steel guide ring with an inner diameter of 10 mm.

The device is prepared by partially pushing one of the cylinders into a guide ring and positioning it on the intermediate anvil fitted with a locating ring. With a measuring spoon, 40 mm^3^ of the prepared high-explosive samples (e.g. crushed and sieved to a particle size of 500–1000 µm) are placed inside the impact device, ensuring that a centre heap is formed. The impact device is then closed with a second steel cylinder by carefully pressing it into the guide ring until it touches the sample. For impact sensitivity testing, assorted drop weights with masses ranging from 0.25 kg to 10 kg are available. The body of each drop weight has two guide grooves, in which it moves between the guide rails. It is equipped with a suspension spigot that arrests the weight in the release mechanism and is further provided with a cylindrical striker, a height marker and the rebound catch for stopping the weight after rebounding from the anvil. Based on the anticipated results (e.g. on the basis of the specific characteristics of the explosive substance undergoing the test), the drop weight is secured in the release mechanism, and the weight is then positioned to the desired height. When the release mechanism is activated, the drop weight is unlocked, and its striking head impacts the upper cylinder of the impact device.

Depending on the characteristics of the tested explosive substance, the mass of the drop weight and drop height (the combined product of which is the impact energy), the sample may or may not initiate upon impact. When evaluating the results, a distinction is made between *no reaction*, *decomposition* (without flame or explosion) and *explosion* (with weak to strong report or inflammation). The *decomposition* and *explosion* can be verified based on several factors, including sound, gas, flame and smoke or via an inspection of the impact device for sooty deposits after removing the upper cylinder. If none of these effects are noticed, an initiation failure (*no reaction*) is registered. Of the three possible types of reactions, both *decomposition* and *explosion* are considered positive test reactions (initiations) according to STANAG test procedures [39]. In our experiments, in addition to audio-visual observations, a decomposition gas detector (MultiRAE model PGM6208) was used to classify the reactions.

The tests were performed at ambient temperatures (i.e. 23.6°C ± 1.4°C), according to the United Nations' ([40] p. 80) recommended test conditions. As the scope of the test method was within the range of −30°C to +80°C, no particular environmental modification was required.

As repeated drops from the same height in a fallhammer will not invariably yield the same result (reaction versus no reaction), the impact sensitivity of an energetic material must be estimated statistically. Hence, the weight is dropped repeatedly from a range of (log) heights $x_{1},\ldots ,x_{n}$ , and for each $x_{i}$ , we observe a binary outcome $y_{i}\in \left\{0,1\right\}$ , where $y_{i}=1$ if a reaction occurred and $y_{i}=0$ otherwise. In accordance with STANAG 4489, the heights are determined according to the Bruceton up-and-down procedure [47], meaning that an initial height $x_{1}$ is chosen for the first drop, and the consecutive heights are chosen inductively by


(2.1) $$\begin{array}{c} x_{i}=\left\{ \begin{array}{ll} x_{i-1}+d & \text{if}\;y_{i-1}=0\\ x_{i-1}-d & \text{if}\;y_{i-1}=1, \end{array}\right. \end{array}$$

for i=2,…,n , where d>0 is the step size of the test, chosen by the operator. That is, we descend one step if a reaction is observed and ascend one step if not. In our experiments, the step size was set as d=0.05 , in accordance with STANAG 4489.

When assessing sensitivity, our primary interest lies in quantiles such as $h_{50}$ , which repesents the height from which there is a 50% probability of a reaction occurring. The median $h_{50}$ is of particular interest, as it is known to correlate with the quantum chemical properties of the energetic material [48].

In addition to point estimates, we also aim to quantify the uncertainty of our results using confidence intervals (CIs). The use of large-sample theory to construct CIs for the Bruceton up-and-down method was verified by Christensen, Stoltenberg, and Hjort [49]. Christensen *et al*. [24] found via simulations that Fieller’s theorem yields the most satisfactory CIs for the quantiles when the Bruceton up-and-down method is employed. As recommended by Christensen *et al*. [24], we used the existence of a bounded 95% CI for $h_{50}$ via Fieller’s theorem as a necessary criterion for terminating our fallhammer experiments. This resulted in most of the datasets comprising more than 30 drops. The fact that 30 drops were not sufficient could allude to the inhomogeneity of the substances tested or statistical model misspecification. Although it would be possible to simply employ the delta method for constructing CIs, as suggested by Dixon and Mood [47], simulation studies consistently show that CIs constructed via Fieller’s theorem are more accurate for sensitivity data (see Christensen *et al*. [24] and the references therein). In particular, the use of Fieller’s theorem does not impact the qualitative conclusions reached in this paper but rather increases the accuracy with which they are derived.

## 3. Results

Prior to all testing, a reference material of recently produced TNT (‘Trinitrotoluene Type 1, Flake’) with a 0.44% content of hexanitrostilbene (HNS), produced by Zaklady Chemiczne ‘NITRO-CHEM’ S.A. in Bydgoszcz, Poland, released for sale following the Certification of Compliance and Analysis on 8 September 2017, was tested. The test of the reference sample revealed an impact sensitivity ( $h_{50}$ ) of 29.8 J, coinciding with the reported value (30 J) as described in STANAG 4489 [39]. The full data from the impact sensitivity tests using the BAM Impact Apparatus are available at Novik and Christensen [50]. Here, we review the main results.

For substance A_1_, we initially aimed to obtain a single reaction with a 5 kg weight, but when this was not achieved, we proceeded to drop a 10 kg weight to increase the impact energy. After the first five drops, we still had no reactions and we therefore decided to execute 10 drops from the maximum height of 100 cm with the 10 kg weight. Out of these, only a single drop caused a reaction. Thus, for this experiment, the maximum likelihood estimators (MLEs) do not exist, and we have highly limited information about the true underlying parameters governing the sensitivity of substance $A_{1}$ . We can, however, assert with relatively high confidence that $h_{50}$ is above 98.07 J, that is, 100 cm with a 10 kg weight.

For substance A_2_, we did not obtain a bounded 95% CI for $h_{50}$ after the first n=30 drops, and we therefore increased the number of drops in increments by 10 at a time until a valid confidence interval was achieved. This happened after n=70 drops. The resulting estimate for $h_{50}$ is 10.99 J, or 22.41 cm with the 5 kg weight. The 95% and 99% confidence intervals for $h_{50}$ is [8.26 J, 13.06 J] and [5.25 J, 14.45 J], respectively. We see that the value of $h_{50}$ is significantly less than 30 J.

For substance B, we decided to stop the experiment after n=30 drops, since this proved to be sufficient for obtaining a bounded 95% CI for $h_{50}$ . From the data, the resulting estimate for $h_{50}$ is 7.52 J, or 15.34 cm with a 5 kg weight. The 95% CI for $h_{50}$ is [3.53 J, 9.25 J]. We did not obtain a 99% CI for $h_{50}$ , since we only did n=30 drops. However, we see that the value is significantly smaller than 30 J.

For substance C, as with substance A_2_, we had not achieved a bounded 95% CI for $h_{50}$ after the first 30 drops, and therefore decided to augment the dataset by increments of 10 drops until this was achieved. After n=50 drops, we had a 95% CI for $h_{50}$ . The resulting estimate of $h_{50}$ is 31.60 J, or 64.42 cm with a 5 kg weight. The 95% and 99% CIs for $h_{50}$ are [28.70 J, 35.90 J] and [27.01 J, 41.06 J], respectively. In particular, we do not have sufficient evidence to reject the hypothesis that $h_{50}=30$ J.

For substance D, since we had not obtained a bounded CI for $h_{50}$ after 30 drops, we increased the number of drops by increments of 10 until this was achieved, at *n* = 70. The resulting estimate of $h_{50}$ is 13.51 J. The accompanying 95% CI for $h_{50}$ is [4.88 J, 19.14 J]. As with substance B, we did not obtain a bounded 99% CI for $h_{50}$ for substance D. Anyhow, we still see that the value for $h_{50}$ is significantly smaller than 30 J.

For substance E, since 30 drops were insufficient for obtaining a bounded 95% CI for $h_{50}$ , we increased the number of drops in increments of 10 until a valid CI was obtained, after n=70 drops. The resulting estimate of $h_{50}$ is 15.37 J, with 95% CI [12.13 J, 19.06 J]. Again, this is significantly smaller than 30 J.

**Figure 4.**
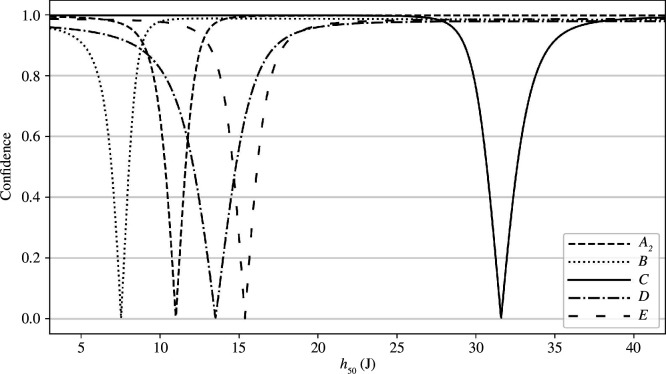
Confidence curves for $h_{50}$ for substances A_2_, B, C, D and E.

To summarize our results graphically, we draw the confidence curves for $h_{50}$ for substances A_2_, B, C, D and E as shown in figure 4. These were drawn using Fieller’s theorem, as explained by Christensen *et al*. [24]. Using these curves, we may obtain all CIs for any confidence level. For example, if we were to calculate where the line y=0.95 intersects these curves, we would recover 95% CIs reported in the previous sections. As we can see, there is a substantial distance between the confidence curve for substance C and the other substances, whose confidence curves overlap more. This reflects how substance C exhibited impact sensitivity in accordance with the existing literature on Amatol (30 J), while all the other substances were significantly more sensitive to impact. Note also how some of the curves are skewed, which reflects the asymmetric confidence intervals reported in the previous sections.

## 4. Summation and discussion

This study demonstrates that Amatols extracted from ERW, with the expected exception of the sample of high moisture content, are still sensitive to impact. For only one of the samples studied, the impact sensitivity coincided with what is recorded in the literature as expected values for Amatol. All the other samples studied were, on the other hand, significantly more sensitive to impact. In the most extreme case, namely substance B, the substance was nearly four times more sensitive than anticipated (the estimate value of $h_{50}$ being only 7.52 J, which is nearly less than a quarter of the expected value of 30 J). Note also that for this substance, we observed reactions with impacts as low as 6.18 J, as shown in Novik and Christensen [50]. The study therefore shows that the impact sensitivity of Amatol high explosives extracted from ageing ERW is susceptible to becoming increasingly sensitive to impact. Earlier studies have shown that explosive compositions containing ammonium nitrate might become sensitized if contaminated and/or exposed to certain environmental factors (e.g. moisture and heat). Albeit all the explosive objects included in this study that showed a significant increase in impact sensitivity were located in cold climatic areas, and their moisture contents were found to be negligible, it cannot be disregarded that these explosives at one point of time have been subjected to heat and/or moisture, or that the explosives could have been contaminated by impurities. Further research into possible variations, resulting from chemical, technical or environmental differences, will be required in order to gain further knowledge on ageing munitions containing Amatol.

Although not able to conclusively trace the origin of the increase in impact sensitivity, our study shows unambiguously that Amatols in ageing munitions can be much more sensitive to impact than previously assumed. This is imperative, as most risk assessments concerning ERW regularly seek to ascertain the societal risks related to ageing munitions. In this regard, particularly the risk of an unintentional explosion of the munitions is of great relevance, as the ordnance is prone to detonate given sufficient stimulus. Such an explosion could be the result of a number of causes. For example, it could occur as a result of an intended act of crime or terrorism, chemical or technical deterioration could cause spontaneous detonation, or it could arise as a result of an accidental or intentional disturbance (e.g. construction work, fishing and recreational activities). Sometimes negligence towards the risks posted by the ERW can result in unauthorized handling of the ordnance, with false reassurance that the explosives do not pose any significant risks [13].

As munitions can remain intact and functional for decades, and even centuries, after the end of hostilities, ERW contamination is generally considered a major threat to societal safety and security. Simultaneously, toxic compounds, including nitroaromatic explosives, are released into the environment by deteriorating munitions, representing an acute ecological and health hazard, resulting in serious environmental pollution problems in several countries and regions worldwide [51,52]. Therefore, the clearance of ERW is a prioritized task in affected areas, and is recognized as a vital risk reduction tool [53]. However, all munitions subject to EOD clearance are, by nature, prone to be handled in one form or another (e.g. moving, relocating, rendering safe). Consequently, if the impact sensitivity of the explosives is in fact significantly higher than previously assumed, this would influence how ERW-related risks are perceived, and form new boundaries for safe and practically feasible disposal techniques.

## 5. Conclusions

In this study, we have analysed the composition of high-explosive substance Amatols that were extracted from ageing ERW. Our results clearly show that the samples studied were significantly more sensitive to impact than one would expect based on the existing literature. A proper understanding of the hazardous properties of ERW is of vital importance, as there exist millions of tonnes of such remnants in nature as unexploded ordnance and munitions disposed of at dumping sites on land, in lakes and at sea. The munitions are continuously deteriorating, resulting in the release of hazardous materials into the environment, potentially posing environmental and societal risks. Moreover, as the explosives deteriorate over time, often resulting from inferior storage conditions or the presence of undesired factors such as moisture and certain metals, the munitions may become increasingly sensitive to external stimuli and susceptible to accidental detonation.

All explosive ordnances subjected to this analysis were initially deemed safe to move and transport by the EOD operative in charge. This decision is based on a number of factors, most predominantly the risks related to detonation *in situ* and their corresponding risk-mitigating actions and those associated with moving or transporting the object to a location that is more suitable for controlled detonation. In this risk assessment, it is imperative to evaluate the technical conditions of the explosive object, including its sensitivity to impact. This is an essential part of assessing whether the object should or could be relocated. However, as this study has demonstrated, these risk assessments were all conducted on the basis of information that has proven to be erroneous. This study has proved that Amatol can potentially have significantly increased impact sensitivity compared to what is listed in most of the literature. Therefore, all risk assessments involving Amatols must account for the fact that handling these substances can pose a greater risk of accidental detonation as a result of increased impact sensitivity than originally assumed.

Furthermore, in addition to Amatol being one of the high-explosive compositions most extensively used up until the end of WWII, several seemingly identical explosive objects were produced with alternating fillings, in which the same object could contain several explosives or explosive compositions. Consequently, we must not only assume that the filling in explosive ordnance containing Amatols can develope increased impact sensitivity, but also, as the exact filling of various ordnance cannot always be verified by external features alone, the risk of increased impact sensitivity must be considered for all explosive ordnance potentially containing Amatols. We, therefore, recommend that EOD operators and other risk assessors must now account for the increase in the impact sensitivity of Amatol in ageing ordnance and factor in this when encountering all munitions potentially containing Amatols. Moreover, as the required number of drops, as described in the relevant standardized methodology of impact sensitivity testing (e.g. as NATO STANAG 4489), was not enough to produce a valid confidence interval in the majority of the experiments, these standards should therefore be revised to include a suitable method for constructing confidence intervals, such as Fieller’s theorem. In particular, no fallhammer test should be terminated until a 95% confidence interval for $h_{50}$ has been obtained.

## Data Availability

Our data are deposited at Dryad Digital Repository [50].

## References

[1] Fedoroff BT , Aaronson HA , Reese EF , Sheffield OE , Clift GD . 1960 Encyclopedia of explosives and related items PATR 2700. (ed. U.S.A.R.a.D. Command ), Picatinny Arsenal, New Jersey: U.S. Army Research and Development Command.

[2] Urbanski T . 1967 Chemistry and technology of explosives, III edn. Oxford: Pergamon Press.

[3] Robertson R . 1920 The research department, woolwich. Nature **105** , 710–712. (10.1038/105710a0)

[4] The War Office [UK] . 1925 Text book of explosives used in the service. The War Office.

[5] The War Office [UK] . 1944 Ammunition inspection guide. TM 9-1904: War Department.

[6] U.S. Army Material Command . 1965 Handbook of foreign explosives. Washington, D.C: US Army Foreign Science and Technology Center.

[7] Albright R . 2012 Cleanup of chemical and explosive munitions: location, identification and environmental remediation, 2nd edn. Massachusetts, U.S: William Andrew.

[8] Hamer M . 2004 The doomsday wreck. See https://www.newscientist.com/article/mg18324615-100-the-doomsday-wreck/

[9] Frey T . 2023 UXO and environmental risk factors impacting EOD operations in German waters. Propellants. Explo. Pyrotec. (10.1002/prep.202300206)

[10] Pfeiffer F . 2012 Changes in properties of explosives due to prolonged seawater exposure. Mar. Technol. Soc. j. **46** , 102–110. (10.4031/MTSJ.46.1.5)

[11] Ford G , Ottemöller L , Bapite B . 2005 Analysis of explosions in the bgs seismic database in the area of beaufort’s dyke, 1992-2004. See https://webarchive.nationalarchives.gov.uk/20121203195642/http://www.mod.uk/NR/rdonlyres/712B6133-E353-4030-9DD0-F677DC3B6F38/0/bgs_beauforts.pdf

[12] Nordaas MTK . 2019 Ammunisjonen kan bli brukt til bomber i tilsiktede handlinger [the ammunition can be used for bombs in intentional acts] Nærnett [Norwegian]. See https://www.nernett.no/artikler/nyhende/sjodumpet-ammunisjonukjente-konsekvenser (accessed 13 October 2019)

[13] Novik GP . 2022 Analysis of samples of high explosives extracted from explosive remnants of war. Sci. Total Environ. **842** , 156864. (10.1016/j.scitotenv.2022.156864) 35752239

[14] Arsenal P . 1943 Study properties of tetrammino cupric nitrate. Dover, N. J: Technical Group, Chemical Department, Research Division, Picatinny Arsenal.

[15] Sunahara GI , Kuperman RG , Lotufo GR , Hawari J , Thiboutot S , Ampleman G . 2009 Introduction. In Ecotoxicology of explosives (eds GI Sunahara , GR Lotufo , RG Kuperman , J Hawari ), pp. 1–3, Boca Raton, FL: CRC Press. (10.1201/9781420004342)

[16] ATSDR . 1995 Toxicological profile for 2,4,6-trinitrotoluene. retrieved from U.S. department of health and human services – agency for toxic substances and disease registry. See https://wwwn.cdc.gov/TSP/ToxProfiles/ToxProfiles.aspx?id=677&tid=125 37647460

[17] Koske D , Goldenstein NI , Kammann U . 2019 Nitroaromatic compounds damage the DNA of zebrafish embryos (Danio rerio). Aquat. Toxicol. **217** , 105345. (10.1016/j.aquatox.2019.105345) 31715477

[18] Koske D , Goldenstein NI , Rosenberger T , Machulik U , Hanel R , Kammann U . 2020 Dumped munitions: New insights into the metabolization of 2,4,6-trinitrotoluene in Baltic flatfish. Mar. Environ. Res. **160** , 104992. (10.1016/j.marenvres.2020.104992) 32907729

[19] Schuster R , Strehse JS , Ahvo A , Turja R , Maser E , Bickmeyer U , Lehtonen KK , Brenner M . 2021 Exposure to dissolved TNT causes multilevel biological effects in Baltic mussels (Mytilus spp.). Mar. Environ. Res. **167** , 105264. (10.1016/j.marenvres.2021.105264) 33725510

[20] Yinon J . 1990 Toxicity and metabolism of explosives. Florida, U.S: CRC Press. (10.1201/9781439805299) See https://www.taylorfrancis.com/books/9781439805299

[21] Maser E , Strehse JS . 2021 Can seafood from marine sites of dumped World War relicts be eaten? Arch. Toxicol. **95** , 2255–2261. (10.1007/s00204-021-03045-9) 33837803 PMC8241755

[22] Novik GP , Sommer M , Abrahamsen EB . 2022 A risk-increasing safety strategy? evaluating the traditional risk mitigating strategy in dealing with dumped ammunition and explosive remnants of war. J. Mil. Hist. Stud. **22** .

[23] Novik GP , Abrahamsen EB , Sommer M . 2023 Improving the decision-making basis by strengthening the risk assessments of unexploded ordnance and explosive remnants of war. Saf. Sci. **160** , 106065. (10.1016/j.ssci.2023.106065)

[24] Christensen D , Unneberg E , Høyheim E , Jensen TL , Hjort NL . 2023 Improved measurements of impact sesitivities of energetic materials. In 25th international seminar on New Trends in Research of Energetic Materials (NTREM).

[25] Urbanski T . 1965 Chemistry and technology of explosives, II edn. Oxford: Pergamon Press.

[26] Gibbs TR , Popolato A (eds). 1980 LASL explosive property data. Los Angeles: University of Calofornia Press. (10.1525/9780520313743)

[27] U.S. Department of the Army . 1984 Military explosives TM 9-1300-214. Department of the Army.

[28] PubChem . 2024 Coumpound summary—Amatol. See https://pubchem.ncbi.nlm.nih.gov/compound/Amatol

[29] Fedoroff BT , Aaronson HA , Clift GD , Reese EF . 1958 Dictionary of explosives, ammunition and weapons (German section), PATR 2510. Dover, New Jersey: U.S. Army Research and Development Command.

[30] Hershkowitz J , Akst I . 1975 A new approach to improving the performance of non-ideal explosives containing ammonium nitrate. Dover, New Jersey.

[31] U.S. War Department . 1945 War department technical manual - ammunition, general, TM 9-1900. Washington: War Department.

[32] Montesi LJ , Menichelli VJ . 1964 Evaluation of ammonium nitrate, aluminium mixture (80/20). White Oak, Maryland: U.S. Naval Ordnance Laboratory.

[33] Johansen SR . 2005 Ammonal fra sola overlevert av kaptein Novik. Analytical Report. Dyno Nobel.

[34] Der Reichsminister der Luftfart . 1942 Die Munition der Flakartillerie, Beschreibung, Teil 1, L.Dv.4402/1. Berlin: Der Reichsminister der Luftfart.

[35] Ordnance Bomb Disposal Center [US] . German artillery projectiles and fuzes. Washington: Ordnance Bomb Disposal Center.

[36] The War Office [UK] . 1944 Handbook of enemy ammunition, Pamphlet no.11. London: His Majesty’s Stationery Office.

[37] U.S. War Office . 1953a German explosive ordnance (bombs, fuzes, rockets, land mines, grenades and igniters) TM 9-1985-2. Washington: United Stated Government Printing Office.

[38] U.S. War Office . 1953b German explosive ordnance (projectiles and projectile fuzes) TM 9-1985-3. Washington: United States Government Printing Office.

[39] NATO . 1999 NATO STANAG 4489 explosives, impact sensitivity tests. North Atlantic Treaty Organization (NATO).

[40] United Nations . 2019 Manual of tests and criteria, 7th rev. ed edn. New York: United Nations.

[41] Rădeanu C , Rus DC , Jitea IC , Miron C , Vasilescu G . 2020 Assessing the impact sensitivity of explosives using the BHF-12A equipment. MATEC Web Conf. **305** , 00011. (10.1051/matecconf/202030500011)

[42] Gruhne MS , Lommel M , Wurzenberger MHH , Szimhardt N , Klapötke TM , Stierstorfer J . 2020 OZM ball drop impact tester (BIT‐132) vs. BAM standard method – a comparative investigation. Propellants Explo. Pyrotec. **45** , 147–153. (10.1002/prep.201900286)

[43] Meyer R , Köhler J , Homburg A . 2005 Explosives. Weinheim: Wiley-VCH Verlag GmbH.

[44] Sućeska M . 1995 Test methods for explosives. New York, NY: Springer. (10.1007/978-1-4612-0797-9)

[45] Monogarov KA , Meerov DB , Fomenkov IV , Pivkina AN . 2023 Energy transferred to energetic materials during impact test at reaction threshold: look back to go forward. Fire Phys. Chem. **3** , 255–262. (10.1016/j.fpc.2022.11.003)

[46] Samseth JØ . 2022 Which bond cleavage is the explosive’s sensitivity most sensitive to? In 51st International Annual Conference of the Fraunhofer ICT.

[47] Dixon WJ , Mood AM . 1948 A method for obtaining and analyzing sensitivity data. J. Am. Stat. Assoc. **43** , 109–126. (10.1080/01621459.1948.10483254)

[48] Jensen TL , Moxnes JF , Unneberg E , Christensen D . 2020 Models for predicting impact sensitivity of energetic materials based on the trigger linkage hypothesis and Arrhenius kinetics. J. Mol. Model. **26** , 65. (10.1007/s00894-019-4269-z) 32130532 PMC7256078

[49] Christensen D , Stoltenberg EA , Hjort NL . 2023 Sequential experimental designs in regression: theory for the bruceton and langlie designs. arXiv. See https://arxiv.org/pdf/2312.13387.pdf

[50] Novik GP , Christensen D . 2023 Data from: Increased impact sensitivity in ageing high explosives: analysis of Amatol extracted from explosive remnants of war. Dryad Digital Repository. (10.5061/dryad.sqv9s4n91) PMC10966400 38545614

[51] Barreto-Rodrigues M , Silva FT , Paiva TCB . 2009 Characterization of wastewater from the brazilian TNT industry. J. Hazard. Mater. **164** , 385–388. (10.1016/j.jhazmat.2008.07.152) 18818021

[52] Luo J , Li Y , Cao H , Zhu Y , Liu X , Li H , Liao X . 2023 Variations of microbiota in three types of typical military contaminated sites: Diversities, structures, influence factors, and co-occurrence patterns. J. Hazard. Mater. **443** , 130290. (10.1016/j.jhazmat.2022.130290) 36335906

[53] Novik GP . 2023 When a safety measure becomes a risk accelerant: removing the option to blast-in-place when clearing explosive remnants of war. J. Conv. Weapons Destr. **27** , 1.

